# Host-Guest Complexation Studied by Fluorescence Correlation Spectroscopy: Adamantane–Cyclodextrin Inclusion

**DOI:** 10.3390/ijms11010173

**Published:** 2010-01-12

**Authors:** Daniel Granadero, Jorge Bordello, Maria Jesus Pérez-Alvite, Mercedes Novo, Wajih Al-Soufi

**Affiliations:** 1 Departamento de Química Física, Facultade de Ciencias, Universidade de Santiago de Compostela, E-27002 Lugo, Spain; E-Mails: daniel.granadero@usc.es (D.G.); jorge.bordello@usc.es (J.B.); m.novo@usc.es (M.N.); 2 Departamento de Química Orgánica, Facultade de Química, Universidade de Santiago de Compostela, E-15782 Santiago de Compostela, Spain; E-Mail: mj.perez.alvite@usc.es (M.J.P-A.)

**Keywords:** fluorescence correlation spectroscopy, host-guest chemistry, adamantane, cyclodextrin

## Abstract

The host-guest complexation between an Alexa 488 labelled adamantane derivative and β-cyclodextrin is studied by Fluorescence Correlation Spectroscopy (FCS). A 1:1 complex stoichiometry and a high association equilibrium constant of *K* = 5.2 × 10^4^ M^−1^ are obtained in aqueous solution at 25 °C and pH = 6. The necessary experimental conditions are discussed. FCS proves to be an excellent method for the determination of stoichiometry and association equilibrium constant of this type of complexes, where both host and guest are nonfluorescent and which are therefore not easily amenable to standard fluorescence spectroscopic methods.

## 1. Introduction

**Figure 1.**
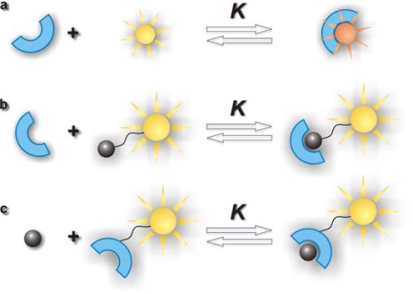
Fluorescent labelling of a host-guest complex (a) inclusion of a fluorescent guest (b) guest with attached fluorophore (c) host with attached fluorophore.

Supramolecular host-guest chemistry describes the formation of molecular complexes composed of small molecules (guests) noncovalently bound to larger molecules (hosts) in a unique structural relationship [1]. Host-guest complexes are of great technological importance and have been extensively studied [2]. Several techniques such as calorimetry, conductivity, pH potentiometry, capillary electrophoresis, and absorption or fluorescence spectroscopy are used to determine their stoichiometry and stability. Among these, fluorescence spectroscopy is widely used, because it is a sensitive and relatively straightforward technique. Standard fluorescence spectroscopy analyzes the variation of a spectroscopic property (quantum yield, spectral shift, lifetime, or anisotropy) of a fluorescent guest or host due to the complexation. A significant variation of any of these parameters requires an intimate participation of the fluorophore in the complexation process, which limits the use of this technique to cases where the fluorophore itself is included as guest (Figure 1a) or is expulsed from the interior of the host by a nonfluorescent guest in a competitive process or where some specific interactions take place. Most technologically interesting host-guest complexes are themselves nonfluorescent and the attachment of a fluorescent label in order to use them in standard fluorescence spectroscopy leads to a dilemma: on one hand, the fluorophore should not interfere in the host-guest complexation under study, but on the other hand a sufficiently strong interaction between fluorophore and host or guest is necessary in order to detect a change in the spectral properties upon complexation. Although many specific solutions have been found, the study of fluorescently labelled host-guest systems by standard fluorescence spectroscopy is still challenging.

Fluorescence Correlation Spectroscopy (FCS) can solve the described problem in a more general way. Instead of the change in the spectral properties FCS analyses the variation in the diffusion coefficient of a fluorophore attached to guest or host due to the increase in the molecular weight upon complexation (see Figures 1b,c) The fluorophore itself need not to interact directly with the host-guest complex except for a common diffusive movement. This relaxes the conditions imposed on the fluorophore which can be selected independently of the specific host-guest system, so that bright and photostable dyes can be attached at convenient positions in guest or host.

**Figure 2.**
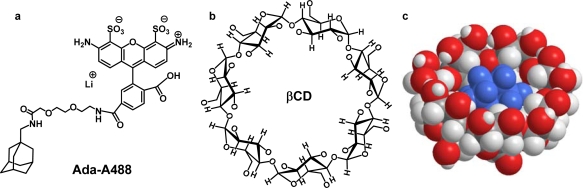
(a) Structure of Ada-A488 (b) Structure of βCD. (c) Sketch of an adamantane-βCD inclusion complex.

FCS is a well established fluctuation correlation method that extracts information about the dynamics of molecular processes from the small changes in molecular concentration or chemical states that arise from spontaneous fluctuations around equilibrium [3]. FCS allows one to study dynamic and photophysical processes that take place in a wide time scale in one and the same experiment. It is a single molecule technique which uses very small sample volumes determined by a confocal setup and nanomolar fluorophore concentrations. FCS is used in a wide range of fields, but surprisingly few applications to the study of host-guest complexation can be found. We studied recently by FCS host-guest dynamics and determined the fast entry/exit rate constants of fluorescent dyes within cyclodextrins [4,5]. In this contribution we will study by FCS the stoichiometry and the stability of the inclusion complex formed between the host β-cyclodextrin (βCD) and the nonfluorescent guest adamantane labelled with Alexa 488 as fluorescent probe (see Figure 2).

Cyclodextrins (CD) are naturally occurring water-soluble toroidally shaped polysaccharides with a highly hydrophobic central cavity that have the ability to form inclusion complexes with a variety of organic and inorganic substrates [6–12]. The three major natural cyclodextrins are α–, β– and γ–CD built up from 6, 7 and 8 glucopyranose units, respectively. CDs are often found as building blocks of supramolecular systems, self-assemblies or chemical sensors [13–18]. The ability of CDs to form inclusion complexes, in which the physicochemical properties of the guest molecules change with respect to the free molecules, has led to a variety of applications [19–25].

Adamantane (tricyclo[3.3.1.1(3,7)]decane, C_10_H_16_) is formed by four cyclohexanes fused to each other in chair conformations achieving a strain free and highly symmetrical stable structure. The adamantyl group is a spherical group with a diameter of 7 Å which perfectly matches the cavity diameter of βCD. Adamantane derivatives form therefore 1:1 inclusion complexes with βCD with high values of the association equilibrium constant, typically between 10^4^–10^5^ M^−1^ [26–31]. Due to their high stability βCD-adamantane complexes have found several important applications both in supramolecular chemistry and in biomedical applications, such as hydrogels [32], affinity biosensors [33], surface-mediated gene delivery [34], cyclodextrin polymer-based particles [35, or supramolecular polymers [36,37].

In this work we demonstrate how FCS can be used to study the inclusion complex formation between βCD and adamantane labelled with the fluorescent probe Alexa 488 (Ada-A488 as shown in Figure 2). We discuss the necessary experimental conditions, determine the stoichiometry and the equilibrium constant and compare the results with those published for similar guests obtained with other methods.

## 2. Theory

### 2.1. Mechanism

The association of the fluorescent guest A and the nonfluorescent host H yielding a fluorescent complex B is treated as a reversible chemical reaction with (association) equilibrium constant *K*:

(1) $$\text{A}+\text{H}\overset{K}{⇌}\text{B}$$

The equilibrium constant *K* is related to the entry (association) (*k*_+_) and exit (dissociation) (*k*_−_) rate constants as follows:

(2) $$K=\frac{k_{+}}{k_{-}}$$

Under conditions where the host concentration [H] is always much higher than that of the fluorescent guest, this concentration coincides with the initial host concentration [H]_0_, and the complexation “reaction” is pseudo-first-order with the relaxation (“reaction”) time *τ_R_* given by:

(3) $$\tau_{\text{R}}=(k_{+}[\text{H}{]}_{0}+k_{-}{)}^{-1}$$

### 2.2. FCS

FCS analyzes the fluorescence intensity fluctuations that are caused by the spontaneous variations in the number of fluorescent molecules in the confocal sample volume due to translational diffusion [5,38–40]. The observed fluorescence intensity fluctuates at a time scale given by the mean residence time of a fluorophore in the sample volume. The intensity fluctuations *δF*(*t*) = *F*(*t*) – <*F*> are analyzed by the normalized temporal autocorrelation function *G*(*t*) as function of the correlation time *τ* as given in Equation (4):

(4) $$G(\tau )=\frac{〈\delta F(t)\;\cdot \;\delta F(t+\tau )〉}{{〈F(t)〉}^{2}}$$

The time dependent part of the correlation function describing pure translational diffusion of a single fluorescent species in and out of a sample volume *G*_D_ is given in Equation (5):

(5) $$G_{\text{D}}(\tau )=\frac{1}{N}{\left(1+\frac{\tau }{\tau_{\text{D}}}\right)}^{-1}{\left(1+{\left(\frac{w_{\text{xy}}}{w_{\text{z}}}\right)}^{2}\frac{\tau }{\tau_{\text{D}}}\right)}^{-\frac{1}{2}}$$

Here a three-dimensional Gaussian sample volume is assumed with radial and axial i/*e*^2^ radii *w*_xy_ and *w*_z_, respectively. *N* is the mean number of fluorescent molecules within the sample volume and *τ_D_* is the translational diffusion (transit) time of the molecules across the sample volume, which is related to the translational diffusion coefficient D by Equation (6) [3,41]. The radius of the sampling volume, *w*_xy_, is determined from a calibration measurement with a reference dye with known diffusion coefficient (in this case rhodamine 123) as described in the Experimental section.

(6) $$D=\frac{w_{\text{xy}}^{2}}{4\tau_{\text{D}}}$$

At higher excitation power the dark triplet state of the dye may be significantly populated and a superimposed fast flickering of the fluorescence intensity may be observed with amplitude *A*_T_ and a time constant *τ*_T_ given by the triplet lifetime of the fluorophore. This leads to an additional exponential term in the correlation function as described in Equation (7):

(7) $$G_{\text{DT}}(\tau )=G_{D}(\tau )\cdot \left(1+A_{\text{T}}\;e^{-\tau /\tau_{\text{T}}}\right)$$

In the case that the exchange of the fluorophore between free and bound states is much faster than the typical transit time of the fluorophore across the sample volume (*τ*_R_ *≪ τ*_D_) these states of the fluorophore will not be seen by FCS as two distinct species, but as a single one with a mean diffusion time *τ̄*_D_. The value of *τ̄*_D_ depends then on the individual diffusion coefficients *D*_f_ and *D*_b_ of free and bound fluorophore and on the molar fractions *X*_x_ = *N*_x_/(*N*_f_ + *N*_b_) of these species:

(8) $${\bar{\tau }}_{\text{D}}=\frac{w_{\text{xy}}^{2}}{4(X_{\text{f}}D_{\text{f}}+X_{\text{b}}D_{\text{b}})}={\left(X_{\text{f}}{\left(\tau_{\text{f}}\right)}^{-1}+X_{\text{b}}{\left(\tau_{\text{b}}\right)}^{-1}\right)}^{-1}$$

The full correlation curve describing translational diffusion of two fluorescent species in fast exchange and a common triplet term is given in Equation (9) where the diffusion term is defined by the mean diffusion time *τ̄*_D_:

(9) $$G_{\text{DT}}(\tau )=\frac{1}{N}{\left(1+\frac{\tau }{{\bar{\tau }}_{\text{D}}}\right)}^{-1}{\left(1+{\left(\frac{w_{\text{xy}}}{w_{\text{z}}}\right)}^{2}\frac{\tau }{{\bar{\tau }}_{\text{D}}}\right)}^{-\frac{1}{2}}\cdot \left(1+A_{\text{T}}\;e^{-\tau /\tau_{\text{T}}}\right)$$

In the case of a fluorescent guest, a 1:1 stoichiometry, and under the conditions that the free host concentration is always much higher than that of the guest, [A] ≪ [H], the mean diffusion time *τ̄*_D_ can be expressed as function of the total host concentration [H]_0_, the equilibrium association constant *K* and the limiting values of the diffusion times of free and bound dye, *τ*_f_ and *τ*_b_, respectively:

(10) $${\bar{\tau }}_{\text{D}}=\frac{\tau_{\text{f}}(1+K{\left[\text{M}\right]}_{0})}{1+\frac{\tau_{\text{f}}}{\tau_{\text{b}}}K{\left[\text{M}\right]}_{0}}$$

The equilibrium association constant *K* can then be determined from a fit to a series of mean diffusion times measured at different host concentrations or directly from a global fit to the series of correlation curves.

## 3. Results and Discussion

**Figure 3.**
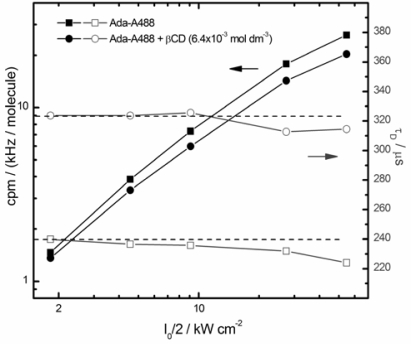
Power series of the FCS signal of Ada-A488 in aqueous solution (squares, [βCD]_0_ = 0 mol dm^−3^) and at high concentration of βCD (circles, [βCD]_0_ = 6.4 × 10^−3^ mol dm^−3^). [Ada-A488] ≈ 10^−9^ mol dm^−3^). Left scale, filled symbols: count rate per single Ada-A488 molecule. Right scale, open symbols: diffusion time of Ada-A488. All data obtained from FCS correlation curves similar to those shown in Figure 4 at different excitation irradiances. Counts per molecule (cpm) is the total detected fluorescence count rate divided by the mean number of molecules in the focus ***N***.

The determination of absolute diffusion coefficients with standard FCS can be affected by several experimental errors [42]. For the determination of the equilibrium constant and the stoichiometry of the complexation only relative values of the diffusion times are needed, but even these may be distorted at high excitation irradiance due to saturation or photodestruction of the fluorophore. The residence time of the complex in the focal volume is longer than that of the free fluorophore, which may increase the photobleaching probability and thus shorten the apparent diffusion times. This in turn flattens the titration curve and leads to an underestimation of the equilibrium constant. Therefore, as a first step the irradiance dependence of the fluorescence count rate and of the diffusion coefficient of free and complexed Ada-A488 has been studied. Figure 3 shows that the registered fluorescence count rate per Ada-A488 molecule (the molecular brightness) (filled black squares) increases linearly at low irradiance but levels off slightly at higher values. The presence of βCD ([βCD] = 6.4 × 10^−3^ mol dm^−3^) has only small influence on the brightness of the fluorophore, with a reduction of about 5% at low and about 25% at highest irradiance. This change may be due to different photobleaching probabilities of free and complexed dye, to polarisation effects in the detection optics, to a change of the refractive index of the solution, or to increased scattering, but it may also be due to some direct interaction between the Alexa 488 chromophore and the adamantane-cyclodextrin complex. However, the Alexa 488 fluorophore is too big to be included into the βCD cavity and no efficient competition with the adamantane inclusion is to be expected.

The diffusion time of Ada-A488 (open squares in Figure 3), increases significantly on the addition of βCD (open circles). At highest irradiance a very similar slight decrease of the diffusion times (of about 5%) is observed in both cases, probably due to some saturation effect. For the titration measurements an irradiance of *I*_0_/2 = 27 kW cm^−2^ was chosen, which is at the upper end of the linear increase of the brightness.

**Figure 4.**
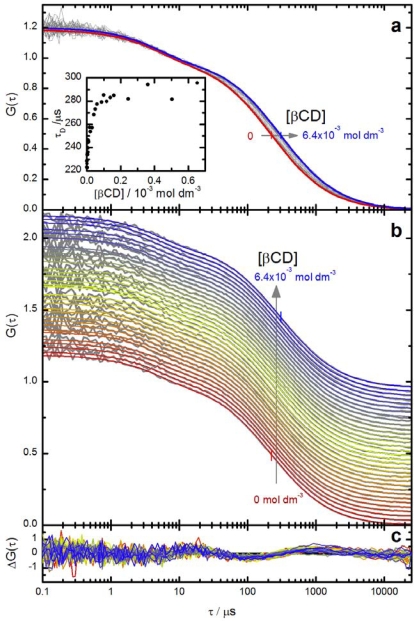
Fluorescence intensity correlation curves *G*(*τ*) of Ada-A488 in the presence of increasing concentrations of βCD ([βCD] = 0 mol dm^−3^ to 6.4 × 10^−3^ mol dm^−3^) in aqueous solution. ([Ada-A488] ≈ 10^−9^ mol dm^−3^). Panel a: normalized experimental correlation curves at increasing βCD concentration (grey curves) and two representative curves from the global fit of Equations. (9) and (10) at [βCD] = 0 mol dm^−3^ (red curve) and [βCD] = 6.4 × 10^−3^ mol dm^−3^ (blue curve) to the correlation curves. The intermediate fit curves are not shown for clarity. Small vertical bars indicate the diffusion time obtained from the fit. Inset: mean diffusion times *τ̄*_D_ as function of βCD concentration determined from individual fits of the Equation (9) to the correlation curves. The highest concentrations are not shown. See also Figure 5. Panel b: stacked representation of the same correlation curves as in panel a. Panel c: weighted residuals from the global fit (vertical scale is arbitrary).

The normalized fluorescence intensity correlation curves *G*(*τ*) of Ada-A488 in water at different βCD concentrations are shown as grey lines in Figure 4. The detected fluorescence intensity is strongly correlated at very short correlation times, but then two decorrelation terms are observed at around 5 μs and 200 μs. The amplitude of the first term increases strongly at higher irradiance (not shown) but is independent of the βCD concentration. It can therefore be safely assigned to the population of the triplet state of Alexa 488. The second term shifts to longer correlation times at increasing βCD concentration and is assigned to the diffusion of the fluorophore in and out of the sample volume. All correlation curves can be well fitted with correlation function *G*_DT_ [Equation (9)] yielding the diffusion times *τ*_D_ shown in the inset of Figure 4a.

**Figure 5.**
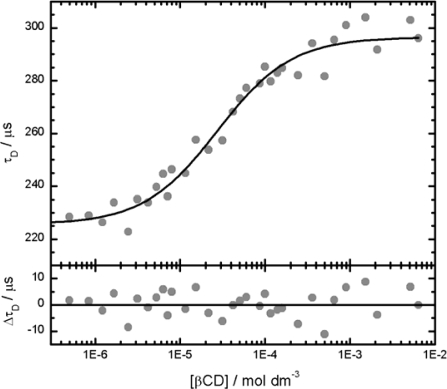
Upper panel: Mean diffusion times *τ̄*_D_ as function of βCD concentration determined from individual fits of correlation function *G*_DT_ [Equation (9)] to the normalized correlation curves of Figure 4. Parameter of the fit as given in the text. The black curve represents the best fit of Equation (10) to *τ̄*_D_ with the parameter *τ*_f_ = 225 ± 2 μs, *τ*_b_ = 297 ± 2 μs, and *K* = (48 ± 7) × 10^3^ mol^−1^dm^3^. Note that due to the logarithmic concentration scale the values at [βCD] = 0 M are not visible in the figure, although they are included in the fit. Lower panel: residuals of the fit.

The strong increase of *τ*_D_ already at very low βCD concentration can not be explained by an increase of the solvent viscosity, which is not significant at these low cyclodextrin concentrations [43]. We interpret these values of *τ*_D_ as mean diffusion times *τ̄*_D_ of the fluorophore in fast exchange between free and bound states as described by Equations (8)–(10). The fit of these mean diffusion times *τ̄*_D_ by the model of a complexation with stoichiometry 1:1 [Equation (10)] is very satisfactory as shown in Figure 5. More precise values of the parameters are obtained by a direct global target fit of the correlation curves by Equations (9) and (10) as shown in Figure 4. The results of this global fit are listed in Table 1, together with calculated values of the diffusion coefficients and the hydrodynamic radii of free dye and the complex.

The limiting diffusion times obtained from the fits to the series of correlation curves were converted to translational diffusion coefficients *D* using Equation (6) with the radial 1/*e*^2^ radius of the sample volume obtained from a calibration with Rhodamine 123 as reference (see experimental section). The uncertainty in this calibration and other systematic errors are not included in the indicated standard deviations. The hydrodynamic radii *R*_h_ of free Ada-A488 and of the complex Ada-A488:βCD were estimated applying the Stokes-Einstein relation [Equation (11)] with the viscosity of water *η* (25 °C) = 0.8905 cP:

(11) $$R_{h}=\frac{kT}{6\pi \eta D}$$

As expected, the free guest Ada-A488 has a higher diffusion coefficient than the complex Ada-A488:βCD. The diffusion coefficient of homogeneous spherical particles is expected to change with the inverse of the cubic root of their molar mass D~R_h_^−1^~M^−1/3^. As shown previously this is well fulfilled for small globular molecules and for inclusion complexes of different cyclodextrins [4]. In this case the ratio between the diffusion coefficients D_b_/D_f_ = 0.74 coincides perfectly with that expected from the ratio of their molar masses (*M_b_*/*M_f_*)^−1/3^ = (2.37)^−1/3^ = 0.75. The absolute values of the diffusion coefficients compare very well with those obtained before for complexes between pyronines and βCD and *γ*CD [4,5].

As can be deduced from the residuals in Figure 5, the precision in the measurement of the diffusion times in these experiments is about *σ*(*τ*_D)_/*τ*_D_ ≈ 2%. This translates to a minimal detectable relative change of the molar mass of the fluorophore from this data of about 20%.

The very high value of the association equilibrium constant *K* = 5.2 × 10^4^ M^−1^ agrees well with that given in the literature for the inclusion of different adamantane derivatives into βCD with values of *K* = 1–10 × 10^4^ M^−1^ [26–31,44].

Finally, the fact that in spite of the high association equilibrium constant a fast exchange of Ada-A488 between free and complexed state is observed indicates that the association rate constant must be similar or even slightly higher than that observed for the dynamics of the association of pyronines to βCD [4,5]. An additional correlation term due to the exchange itself is not observed as the fluorophore does not change its brightness upon complexation.

## 4. Experimental Section

### 4.1. Materials

βCD (Sigma-Aldrich) (*M* = 1134.98 g mol^−1^) was used as delivered. βCD was checked for fluorescence impurities and was found to be clean enough for classical fluorescence measurements and for FCS experiments. Water was purified with a Milli-Q system. The synthesis of the Ada-A488 compound (*M* = 825.88 g mol^−1^) is described in Section 4.4.

### 4.2. Sample Preparation

Stock aqueous solutions of βCD were freshly prepared with a concentration of about 8 × 10^−3^ mol dm^−3^. Stock solutions of Ada-A488 were prepared as follows: the solid compound Ada-A488 was first dissolved in ethanol in order to facilitate its solubilisation. Then, an aliquot of this solution was diluted 1,000 times in 0.1 mol dm^−3^ phosphate buffer to adjust the pH at 6. The concentrations of Ada-A488 in these stock solutions were still 25-fold higher than that necessary for the FCS measurements (approximately 10^−9^ mol dm^−3^). The FCS samples were finally prepared by dilution of a constant volume of the corresponding Ada-A488 stock together with different volumes of the βCD stock solution and addition of water to adjust to a certain total volume. All these volumes were weighed so that concentration corrections could be performed. Special care was taken in order to avoid any possible contamination of the samples with fluorescent impurities. At the highest βCD concentrations a slight turbidity was observed in the samples, which explains an additional small very slow term in the correlation curves.

### 4.3. FCS Measurements

The confocal epi-illuminated setup used for the FCS measurements is similar to that described elsewhere [4,45]. A 40 μL drop of each sample was deposited on a borosilicate coverslip (Menzel Gläser, NO. 1 DE). The samples were excited by the continuous linearly polarized light of a 489 nm laser diode (Becker&Hickl, BDL-485-SMC, DE) coupled to a monomode optical fiber (Point-Source, kineFLEX-P-1-S-405-0.7, UK). The light output of the fiber was collimated (Schäfter&Kirchhoff, 60FC-4-6,2-01-DI, DE), spectrally cleaned (Semrock, Brightline HC 482/18, US), redirected by a dichroic mirror (Semrock, Brightline BS R488, US) and focused into the sample by a high aperture microscope objective (Olympus, UPLSAPO 60xW/1.20, water immersion) mounted in an inverted microscope (Olympus, IX-71). The fluorescence was collected by the same objective and then refocused through the dichroic mirror onto a pinhole (Thorlabs, ∅ = 50 μm, US) in the image plane. The light passing the pinhole was collimated, then split into two beams by a nonpolarizing beamsplitter cube (Newport, 05BC17MB.1, US) and each focused onto avalanche photodiodes (MPD50CTC APD, ∅ = 50 μm, MPD, Italy). Band-pass filters (Semrock, Brightline HC 525/45, US) in front of the detectors discriminated fluorescence from scattered laser light. Both output signals were processed and stored by TCSPC-modules (SPC 132, Becker & Hickl GmbH, Berlin, Germany). Correlation curves were calculated with a fast home-built routine that runs under LabVIEW (National Instruments) [45]. Typically 20 million photons were collected for each correlation curve with count rates around 50 kHz. All measurements were made at stabilized temperature, 25.0 ± 0.5 °C. The excitation power as measured in the focus of the microscope objective by a power meter (Thorlabs, PM30-120, US) was typically 240 μW, corresponding to a mean irradiance of *I*_0_/2 = *P*/(*π*·*ω*_xy_^2^) = 27 kW cm^−2^, assuming a Gaussian intensity distribution along the optical axis. *P* is the excitation power in the sample) [46].

The focal area and the detection volume were calibrated with Rhodamine 123 in aqueous solution at low irradiance yielding a radial 1/*e*^2^ radius of *ω*_xy_ = 0.53 μm. The value of *D*_R123_ = (4.6 ± 0.4) × 10^−10^ m^2^s^−1^ is estimated from recent PFG-NMR [47] and dual-focus FCS [48] data. The diffusion coefficients are given for 25 °C. All given uncertainties correspond to one standard deviation from the fits and do not include calibration errors.

Series of FCS curves measured at different host concentrations were analyzed by global “target” analysis programmed in OriginPro 8.0 (OriginLab Corporation, US). An empirical weighting function was used in order to take into account the strong variation of the noise in the FCS curves.

### 4.4. Synthesis of the Ada-A488 Compound

#### Preparation of compound 1 (see Scheme 1)

1-adamantanemethylamine (85.8 mg, 0.52 mmols) was dissolved in dry DCM (10.4 mL) and Fmoc 8-amino-3,6-dioxaoctanoic acid (200mg, 0.52 mmols), HATU (217 mg, 0.57 mmols) and DIEA (546 mL, 3.12 mmols) were successively added.

After 1 h stirring at rt, the solution was poured into a separation funnel and washed with HCl (5%) and NaHCO_3_ (sat). The organic layers were dried over Na2SO4, filtered and concentrated under reduce pressure, providing a yellow oil that when purified by flash chromatography (2–4% MeOH in DCM) gave (**1**, 222 mg) of the compound as a white foam [80%, Rf = 0.5 (5% MeOH in DCM)].

#### Preparation of compound 2

A solution of Fmoc 8-amino-3,6-dioxaocta-methyladamantane amide (**1**, 25 mg, 0.047 mmols) in piperidine-DCM mixture (1:4, 0.5 mL) was stirred at rt for 20 min, the solvent was removed *in vacuo*, and the residue was dissolved in DCM. This solution was washed with NaOH (1M). The organic phase was concentrated and dissolved in H2O. The resulted solution was centrifugated and the supernatant was liofilized giving 8-amino-3,6-dioxaocta-methyladamantane amide (**2**, 11 mg) as a yellow oil which was used without further purification [75%, Rt = 15.3 min (Eclipse Inertsil analitic column, 50–80 % MeOH 0.1%TFA in H2O 0.1%TFA in 19 min)].

^1^**H NMR** (CD_3_CN, 250.13 MHz, d): 7.04–6.85 (m, 2H, NH_2_), 3.9 (s, 2H, CH_2_ ester), 3.65–3.52 (m, 6H, CH_2_ ether), 3.01–2.89 (bs, 2H, CH_2_ amine), 2.83 (d, *J =* 6.57 Hz, 2H, CH_2_ amide), 1.72–1.47 (m, 7H, CH and CH_2_ Ad), 1.4 (s, 6H, CH_2_ Ad).

#### Preparation of compound 4

8-amino-3,6-dioxaocta-methyladamantane amide (**2**, 0.35 mg, 1.12 mmols) and Alexa Fluor 488 carboxylic acid succinimidyl ester (mixed isomers) (**3**, 0.2 mg, 0.31 mmols) were dissolved in dry DCM (500 mL) and dry DMF (20 mL), DIEA (1 mL, 5.5 mmols) was added and the mixture was stirred under argon for 1 h. The crude was purified by HPLC, affording (**4**, 0.15 mg) of compound as a pink solid [58%, Rt = 15 min and 16min for 2 isomers (Eclipse Inertsil analitic column, 50–80% MeOH 0.1%TFA in H_2_O 0.1%TFA in 19 min)].

**MS (MALDI-TOF**) [m/z (%)]: 825 ([M^−^], 100), 779.4 (8), 604.2 (16).

#### Abbreviations Used

CD_3_CN: Deuterated acetonitrile; DCM: Dichloromethane; DIEA: Diisopropylethylamine; DMF: dimethylformamide; Fmoc: Fluorenylmethoxycarbonyl; HATU:O-(7-azabenzotriazol-1-yl)-1,1,3,3-tetramethyluronium hexafluorophosphate; ^1^H NMR: Proton nuclear magnetic resonance; HPLC: High Performance Liquid Chromatography; MALDI-TOF: Mass Spectrometry of Laser Desorption/Ionization-Time of Flight; bs: broad singlet; d: doublet; m: multiplet; s: singlet; rt: room temperature.

#### Suppliers

Fmoc 8-amino-3,6-dioxaoctanoic acid: Commercially available from Bachem; Alexa Fluor 488 carboxylic acid succinimidyl ester (mixed isomers): Commercially available from Molecular Probes.

## 5. Conclusions

Fluorescence correlation spectroscopy has been proved to be an excellent method for the determination of the stoichiometry and the association equilibrium constant of host-guest complexes with fluorescently labelled guests. FCS requires only that guest or host can be fluorescently labelled and that the complexation increases sufficiently the molar mass of the fluorescent species. FCS needs only minimal amounts of host and guest, is fast and relatively straightforward, as long as the experimental conditions are carefully selected. All commercially available systems allow this type of measurements.

A very high value of the association equilibrium constant between Ada-A488 and βCD was determined, which agrees well with that given in the literature for the inclusion of similar adamantane derivatives into βCD.

## Figures and Tables

**Scheme 1.**
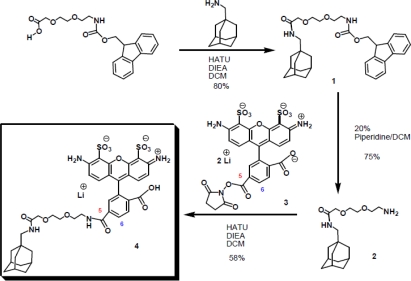
Synthesis of the Ada-A488 compound (4).

**Table 1.** Results of the global target fit of Equations (9) and (10) to the correlation curves shown in Figure 4 and calculated values. All values at 25.0 ± 0.5 °C.

	**Ada-A488 + βCD**
*K*/10^3^ M^−1^	52 ± 2
*τ*_f_/ms	0.222 ± 0.002
*τ*_b_/ms	0.300 ± 0.002
*A_T_*	0.20
*τ*_T_/μs	4.8

*D*_f_/10^−10^ m^2^s^−1^	3.15 ± 0.30
*D*_b_/10^−10^ m^2^s^−1^	2.33 ± 0.20
*R*_h,f_/Å	7.8 ± 0.7
*R*_h,b_/Å	10.5 ± 0.9
